# Herbal Supplements: Can They Cause Hypomania?

**DOI:** 10.7759/cureus.13476

**Published:** 2021-02-21

**Authors:** Sana Elham Kazi, Rusina Karia, Luba Leontieva

**Affiliations:** 1 Psychiatry, State University of New York Upstate Medical University, Syracuse, USA; 2 Psychiatry, State University of New York Upstate Medical University, Syracuse, USA

**Keywords:** herbal supplements, hypomania, hyponatremia, cannabis use

## Abstract

Herbal medication use is prevalent and increasing in the general population. A comprehensive review of complementary and alternative medicine use including herbal medications and supplements is often overlooked by physicians. Patients generally believe that all herbal products are safe without any side-effects. Herbal medications may have complex pharmacodynamics and can be associated with various psychiatric symptoms. The general population, as well as physicians, may be unaware of the risks and side-effects associated with herbal supplement use and further research may be needed. The objective is to describe a case report of acute onset of symptoms of hypomania associated with the increasing use of herbal supplements. A 49-year-old man developed symptoms of hypomania after a two-month history of daily use of a combination of more than 25 herbal supplements and daily cannabis use. Hypomania symptoms were temporally associated with the use of multiple herbal supplements that included ginseng. We recommend that a thorough history of medication use including herbal supplements and other alternative medications and a collateral report from family members and other providers including herbalists be obtained on all patients presenting with psychiatric symptoms. Further research is needed to identify the pharmacodynamics, risks, and adverse effects, and drug and food interactions of each herb.

## Introduction

Eighty percent of the world population relies on traditional medicine for their primary health care needs according to a report by the World Health Organization (WHO) [1]. There has been a steady ascent in the use of herbal supplements in medicine, with sales reaching nearly US$9 billion in 2018 in the United States, which marks its highest growth in supplement sales since 1998 [2]. Based on the National Ambulatory Medical Care Survey Physician Induction Interview conducted in 2012 (n = 5,622), out of all the complementary health approaches, herbal/nonvitamin supplements were recommended 26.5% times by the physicians [3].

Complementary and alternative medication use is common in people with psychiatric illnesses. In a survey of complementary and alternative medicine usage among psychiatric inpatients (n = 82) it was found that 63% used at least one complementary and alternative medication modality within the previous 12 months, including 44% who used herbal medications [4].

Even though there is a widespread use of herbal medications, individuals do not inform their physicians of this history of medication use, nor do many physicians routinely obtain this history from their patients [5-6]. People presume that herbal supplements are safe because they are natural products, and many physicians are unaware of the potential risks and side-effects of these herbal medications. 

In the United States, herbal supplements are regulated by the U.S. Food and Drug Administration (FDA) as food products. They are not regulated as strictly as prescription drugs or over-the-counter (OTC) drugs. Supplements fall under the category of dietary supplements and therefore are not subject to the phases of clinical testing. Scientific evidence on the efficacy and safety of herbal supplements is sparse as there are many challenges and a lack of clear guidelines to conduct research on herbal products. Diagnostic And Statistical Manual Of Mental Disorders, Fifth Edition (DSM-5) introduced the diagnostic category of “substance/medication-induced bipolar and related disorder" [7]. In order to make this diagnosis, a temporal association must be established between the use/withdrawal of substances or medications and the occurrence of mania. We report an unusual case of a patient who presented with symptoms of hypomania associated with the use of more than 25 herbal supplements at the same time.

## Case presentation

A 49-year-old man with a history of depression for nine years, treated with escitalopram, presented to the emergency department with complaints of anger outbursts, insomnia, difficulty with short-term memory, decreased attention span, dizziness, and a herpes outbreak on his lips. Collateral information was obtained from the patient’s wife and mother. The patient's personality and behavior change started about two months ago. He was noted to have anger outbursts, mood swings, excessive crying, talkativeness, flight of ideas, grandiosity, distractibility, indiscretion, hyperactivity, and decreased sleep with subsequent worsening of these symptoms. The patient’s erratic and disorganized behavior had worsened about 10 days before he came to the Emergency Department. The patient was noted by the family to be vigorously cleaning the house, boarding up the attic windows, having post-it notes all over the house, and driving impulsively to various locations. He was also emotionally and verbally abusive towards his wife. Loss of 20 pounds of weight over the past 10 months and increase in social anxiety was also noted. He had been working from home for the last two years. As per the patient and the patient’s family, these symptoms had never occurred before. There was no family history of mental health conditions. The patient denied any suicidal ideation, intent, or plan and denied any auditory or visual hallucinations. He reported a three-year history of myalgias and cannabis use to help with the pain. There was a significant increase in the frequency of cannabis usage for the past three months. He had also recently changed his supplier from a licensed dispensary in MA to a local street dealer. On further evaluation of the patient’s medication history, the patient reported that he had also been taking herbal medications from a licensed acupuncturist for the past two months for his myalgias. He was consuming unknown quantities of these supplements. The patient had a history of alcohol use in the past and had been abstinent since 2003. The patient's father had a history of Alzheimer's dementia and alcohol use disorder. There was no other family history of psychiatric disorders. 

The patient’s consumption of multiple herbal medications along with daily use of possibly tainted cannabis coincided with the onset of hypomanic symptoms. A complete list of the herbal supplements taken by the patient over the past two months (except three medications, that were taken during the first week only) is shown in Table 1.

**Table 1:** Herbal supplements.

Binomial name	Herbal supplement
Chai Hu	Bupleurum Root
Ban Xia (Fa)	Pinellia Rhizome (Processed)
Fu Ling	Poria
Gui Zhi	Cinnamon Twig
Huang Qin (Jiu)	Chinese Skullcap (Processed)
Da Zao	Jujube Fruit
Sheng Jiang	Ginger
Bai Shen	Ginseng Root
Long Gu (Sheng) – The first week only	Dragon's Bone (Bovine Bone)
Mu Li (Sheng) – The first week only	Oyster Shell
Da Huang	Rhubarb
Zhi Qiao (Fu Chao)	Bitter Orange (Processed)
Bai Shao	White Peony Root
Chuan Xiong	Chuanxiong Rhizome
Xiang Fu (Cu Zhi)	Cyperus Rhizome (Processed)
Gan Cao (Mi)	Licorice Root (Processed)
Bai Zhu	White Atractylodes Rhizome
Pao Jiang	Ginger (Blast-Fried)
Bo He	Mint Herb
Gan Cao (Mi)	Licorice Root (Processed)
Niu Xi (Huai)	Achyranthes Root
Dai Zhe Shi (Duan) – The first week only	Hematite (Processed)
Gui Ban (Cu)	Tortoise Plastron (Processed)
Xuan Shen	Scrophularia Root
Tian Dong	Asparagus Root
Yin Chen	Virgate Wormwood Herb
Mai Ya	Barley Sprout
Chuan Lian Zi (Chao)	Toosendan Fruit (Processed)

In the emergency department, the patient was noted to have hypotonic hyponatremia with an initial sodium level of 125 and low serum osmolality of 257 with hypochloremia with chloride level of 94 and mildly elevated creatinine kinase level of 913. The toxicology screen was negative except for cannabinoids. Head CT was negative. The patient received 1 liter of the saline bolus in the emergency room and his sodium level subsequently improved to 132 and creatinine kinase level to 508.

Given that the patient’s behavior had become increasingly disorganized with symptoms of hypomania and concern for the patient’s ability to adequately care for himself at home, he was admitted to the inpatient psychiatric unit.

During the hospitalization, the patient was started on olanzapine and the dose of escitalopram was reduced from 20 mg to 15 mg. Throughout the patient’s hospitalization, he was pleasant and cooperative and stayed in good behavioral control. He did not require any emergent medications, restraints, or seclusions. Over the course of hospitalization, the patient reported improvement in the presenting symptoms including insomnia, flight of ideas, irritability, memory loss, poor concentration, and distractibility. There was an improvement in sodium level to 140 and chloride level to 103. B1, B6, and B12 levels were normal and Lyme and syphilis serologies and heavy metal panel were negative. MRI brain was negative as well. The patient was then discharged home with olanzapine and escitalopram. 

## Discussion

Symptoms of hypomania can be associated with the use of herbal supplements, cannabis use, hyponatremia, and treatment-emergent hypomania associated with the use of escitalopram.

The above-mentioned patient developed symptoms of hypomania only after the start of herbal medications with no prior episodes. In our review of the literature on herbal medication use associated with psychiatric symptoms, it was notable that the symptoms that our patient presented resembled the symptoms and associations with herbal medicine use as mentioned in the literature.

Various case reports have been reported with the use of herbal supplements and symptoms of mania and hypomania. Bostock et al did a systematic review and quality assessment of case reports on mania associated with herbal medications [8]. Fourteen of these case reports showed an association of manic symptoms with the use of St. John's wort (Hypericum perforatum), five of these with the use of Ginseng (Panax ginseng), four of these with the use of Brindleberry (Garcinia cambogia), three of these with the use of Ma huang (Ephedra sinica), two with herbal slimming pills and two with Herbalife products and one with Hydroxycut, horny goat weed (Epimedium grandiflorum), herbal body tonic, celery root (Apium graveolans) and with an herbal mixture. A study done by Ssempijja et al in Uganda found consumption of some raw herbal medicines associated with increased carcinogenic risk [9]. Izzo et al concluded that there are serious clinical consequences on interactions between prescribed drugs and herbal medicines [10]. With more patients using herbal supplements, clinicians should have information and be updated on them. ConsumersLab.com is a website that can be used by clinicians to find information on herbal supplements. It can provide information about the side effects, uses, and the several brands of a specific supplement.

Potential harms of the use of cannabis in patients with psychotic and mood disorders have been documented in the literature [11]. Adolescent cannabis use may be an independent risk factor for future hypomania, and the nature of the association suggests a potential causal link [12,13]. There is also evidence that premorbid cannabis use predicts the development of bipolar disorder [14]. Cannabis use was also associated with a worsened Young Mania score and may worsen the occurrence of manic symptoms in those diagnosed with bipolar disorder and may also act as a causal risk factor in the incidence of manic symptoms [11,15]. Even though the patient has been using street-purchased cannabis of unknown quantity for the past three years, the patient's reported increase in cannabis use may also have contributed to the symptoms of hypomania. 

Treatment-emergent mania/hypomania is an infrequent event, occurring in 2.3% of patients treated for major depression [16]. Given that the patient was on the same dose of Escitalopram for the past seven years, this is a very unlikely cause for the patient’s sudden onset of hypomania symptoms. 

With regards to the association of hyponatremia with hypomania, very few case reports have been reported. One was a case report of recurrent mania in a patient with prior history of bipolar disorder type 1 with recurrent hospital admissions about four times within three weeks presenting with hyponatremia and with manic symptoms during each medical admission [17]. The other one was a case report of manic-psychotic symptoms possibly induced by hyponatremia. Hyponatremia was believed to be induced by excessive water intake during a period of fasting in the context of a wellness practice [18].

The above-mentioned patient’s hypomania symptoms occurred in the context of starting a concoction of herbal medications including the use of Ginseng.

## Conclusions

The main conclusion from the present case report is the importance of understanding which herbal supplements a patient is taking and what potential adverse reactions these supplements could cause. In our case, the previously high-functioning man became acutely manic that caused not only severe functional impairment (job and marital problems), but a dangerous physiological and emotional impairment - i.e., weight loss, hyponatremia, elevated CK, paranoia, agitation, and sleeplessness. We recommend that a thorough history of medication use including herbal supplements and other alternative medications, and a collateral report from family members and other providers including herbalists be obtained on all patients presenting with psychiatric symptoms. Further research is needed to identify the site, mode, and mechanism of action of commonly used herbal supplements in combination and as monotherapy. More information is required on the desired effects, adverse effects, and interactions of herbs with each other, with prescription drugs and OTC medications, and with food. The scientific community should come together to make policies on conducting clinical trials and research on herbal supplements as their use and popularity are increasing among our patients. The limitation of the current case report is the unknown contribution of the street-purchased cannabis that this patient was consuming along with the herbal supplements.
